# Bile Acid–Drug Interaction via Organic Anion-Transporting Polypeptide 4C1 Is a Potential Mechanism of Altered Pharmacokinetics of Renally Excreted Drugs

**DOI:** 10.3390/ijms23158508

**Published:** 2022-07-31

**Authors:** Minami Yamauchi, Toshihiro Sato, Ayana Otake, Masaki Kumondai, Yu Sato, Masafumi Kikuchi, Masamitsu Maekawa, Hiroaki Yamaguchi, Takaaki Abe, Nariyasu Mano

**Affiliations:** 1 Faculty of Pharmaceutical Sciences, Tohoku University, Sendai 980-8578, Japan; minami.yamauchi.t1@dc.tohoku.ac.jp (M.Y.); masafumi.kikuchi.b2@tohoku.ac.jp (M.K.); m-maekawa@tohoku.ac.jp (M.M.); mano@hosp.tohoku.ac.jp (N.M.); 2 Department of Pharmaceutical Sciences, Tohoku University Hospital, Sendai 980-8574, Japan; ayana.otake.d1@tohoku.ac.jp (A.O.); masaki.kumondai.d5@tohoku.ac.jp (M.K.); yu.sato.e7@tohoku.ac.jp (Y.S.); 3 Department of Pharmacy, Yamagata University Hospital, Yamagata 990-9585, Japan; hiroaki.yamaguchi@med.id.yamagata-u.ac.jp; 4 Graduate School of Medical Science, Yamagata University, Yamagata 990-9585, Japan; 5 Division of Nephrology, Endocrinology, and Vascular Medicine, Graduate School of Medicine, Tohoku University, Sendai 980-8574, Japan; takaabe@med.tohoku.ac.jp; 6 Division of Medical Science, Graduate School of Biomedical Engineering, Tohoku University, Sendai 980-8579, Japan; 7 Department of Clinical Biology and Hormonal Regulation, Graduate School of Medicine, Tohoku University, Sendai 980-8575, Japan

**Keywords:** OATP4C1, bile acids, lithocholic acid, bile acid–drug interaction, liver disease, renally excreted drugs

## Abstract

Patients with liver diseases not only experience the adverse effects of liver-metabolized drugs, but also the unexpected adverse effects of renally excreted drugs. Bile acids alter the expression of renal drug transporters, however, the direct effects of bile acids on drug transport remain unknown. Renal drug transporter organic anion-transporting polypeptide 4C1 (OATP4C1) was reported to be inhibited by chenodeoxycholic acid. Therefore, we predicted that the inhibition of OATP4C1-mediated transport by bile acids might be a potential mechanism for the altered pharmacokinetics of renally excreted drugs. We screened 45 types of bile acids and calculated the IC_50_, *K*_i_ values, and bile acid–drug interaction (BDI) indices of bile acids whose inhibitory effect on OATP4C1 was >50%. From the screening results, lithocholic acid (LCA), glycine-conjugated lithocholic acid (GLCA), and taurine-conjugated lithocholic acid (TLCA) were newly identified as inhibitors of OATP4C1. Since the BDI index of LCA was 0.278, LCA is likely to inhibit OATP4C1-mediated transport in clinical settings. Our findings suggest that dose adjustment of renally excreted drugs may be required in patients with renal failure as well as in patients with hepatic failure. We believe that our findings provide essential information for drug development and safe drug treatment in clinics.

## 1. Introduction

In clinical practice, liver and renal functions are used as indices for dose determination of liver-metabolized and renally excreted drugs, respectively. Nevertheless, adverse events occur due to the elevation of plasma drug concentrations, even after considering the patients’ conditions, including the liver and kidney function tests [1]. One of the reported causes is uremic toxins which accumulate in the blood of patients with renal failure, thus affecting hepatic drug transport and metabolism [2,3] and altering the dynamics of liver-metabolized drugs in patients with renal failure.

However, only limited reports have described the mechanism underlying altered dynamics of renally excreted drugs in patients with liver failure. In these patients, the plasma concentration of bile acids significantly increases. Renal drug transporters in the proximal tubular cells are essential for the elimination of some renally excreted drugs [4]. The expressions of renal transporters such as organic anion transporter (OAT) 1, OAT3, multidrug resistance-associated protein (MRP) 2, and MRP4 were altered in a cholestatic model [5,6,7,8]. However, the effect of bile acid–drug interaction (BDI) on renal drug transporters has not been investigated.

Bile acids, the major components of bile, are amphipathic steroidal molecules synthesized from cholesterol [9] that facilitate the absorption of lipids, lipid-soluble nutrients, and drugs [9]. Bile acids also play a critical role in essential physiological functions such as glucose metabolism [10], cholesterol metabolism [11,12,13,14], cellular immunity [15,16], and energy homeostasis [17]. The dynamics of bile acids are regulated by the enterohepatic circulation, in which they are synthesized in the hepatocytes, excreted into the intestine via the bile duct, reabsorbed in the ileum, and returned to hepatocytes via the portal vein. Approximately 95% of bile acids remain in the enterohepatic circulation; however, bile acids that escape from reabsorption in the ileum are lost in the feces or they are minimally transferred to the systemic circulation and excreted into the urine by the kidney [18]. The deposition of bile acids is regulated by transporters such as Na^+^-taurocholate cotransporting polypeptide (NTCP) [19,20,21], organic anion-transporting polypeptide (OATP) 1B1 [19,20,21], OATP1B3 [19,20,21], bile salt export pump [22,23], apical sodium-dependent bile acid transporter [21,24,25], and organic solute transporter α/β [26,27]. Recently, glycochenodeoxycholic acid 3-sulfate (GCDCA-3S) has been reported as a marker of drug interactions with OATP1B1 and OATP1B3 [28]. Since bile acids interact with several drug transporters, BDIs on renal drug transporters were expected.

OATP4C1 is the only member of the OATP family expressed in the kidneys. It localizes to the basolateral membrane of the renal proximal tubular cells and is involved in the excretion of thyroid hormone [29], cyclic adenosine monophosphate [29], uremic toxins [30,31,32,33], cardiac glycosides (ouabain and digoxin) [29,34,35], methotrexate [29], and sitagliptin [36] into the urine. We have previously shown that 10 drugs, namely, nicardipine, spironolactone, fluvastatin, crizotinib, levofloxacin, clarithromycin, ritonavir, saquinavir, quinidine, and verapamil, strongly inhibited OATP4C1 [37]. Moreover, OATP4C1 is responsible for the uptake of remdesivir in the renal proximal tubular cells [38]. Thus, OATP4C1 is recognized as a new player in drug handling within the kidneys. Since Yamaguchi et al. [39] reported that the bile acid chenodeoxycholic acid (CDCA) inhibits OATP4C1-mediated transport, we predicted that other bile acids might interact with OATP4C1. Thus, we hypothesized that the altered dynamics of renally excreted drugs in patients with liver failure may be attributed to the interaction of bile acids with drug transporters, especially OATP4C1.

The aim of our study is to assess the effect of bile acids on OATP4C1-mediated transport to clarify the mechanisms underlying the altered dynamics of renally excreted drugs in patients with liver failure. The findings of this study will aid in dose adjustment of renally excreted drugs not only for patients with renal failure but also for those with liver failure.

## 2. Results

### 2.1. Screening of the Inhibitory Effect of Bile Acids on OATP4C1-Mediated Transport

**Figure 1:**
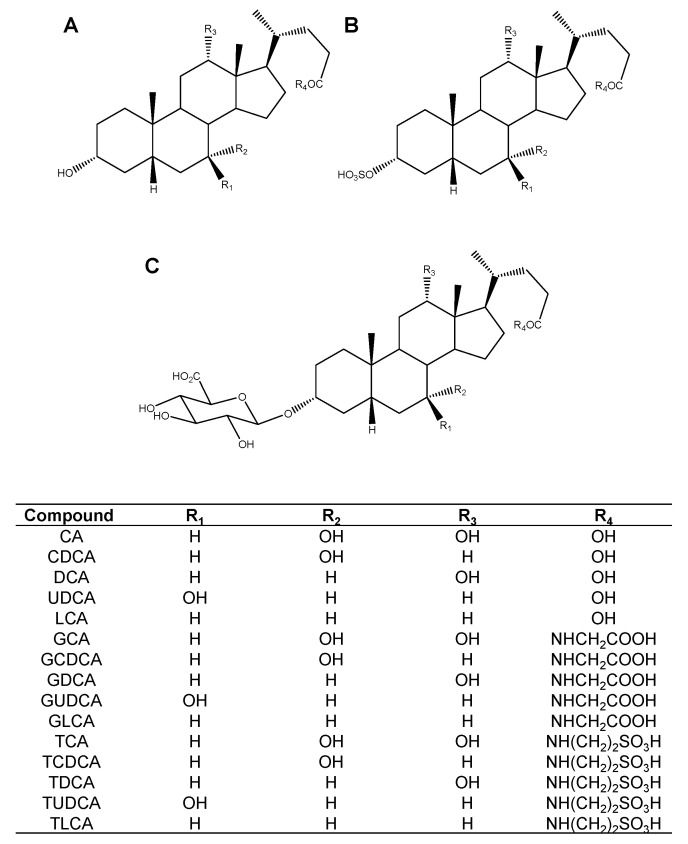
Chemical structures of (**A**) unconjugated, glycine-conjugated, and taurine-conjugated bile acids, (**B**) bile acid 3-sulfates, and (**C**) bile acid 3-glucuronides.

**Figure 2:**
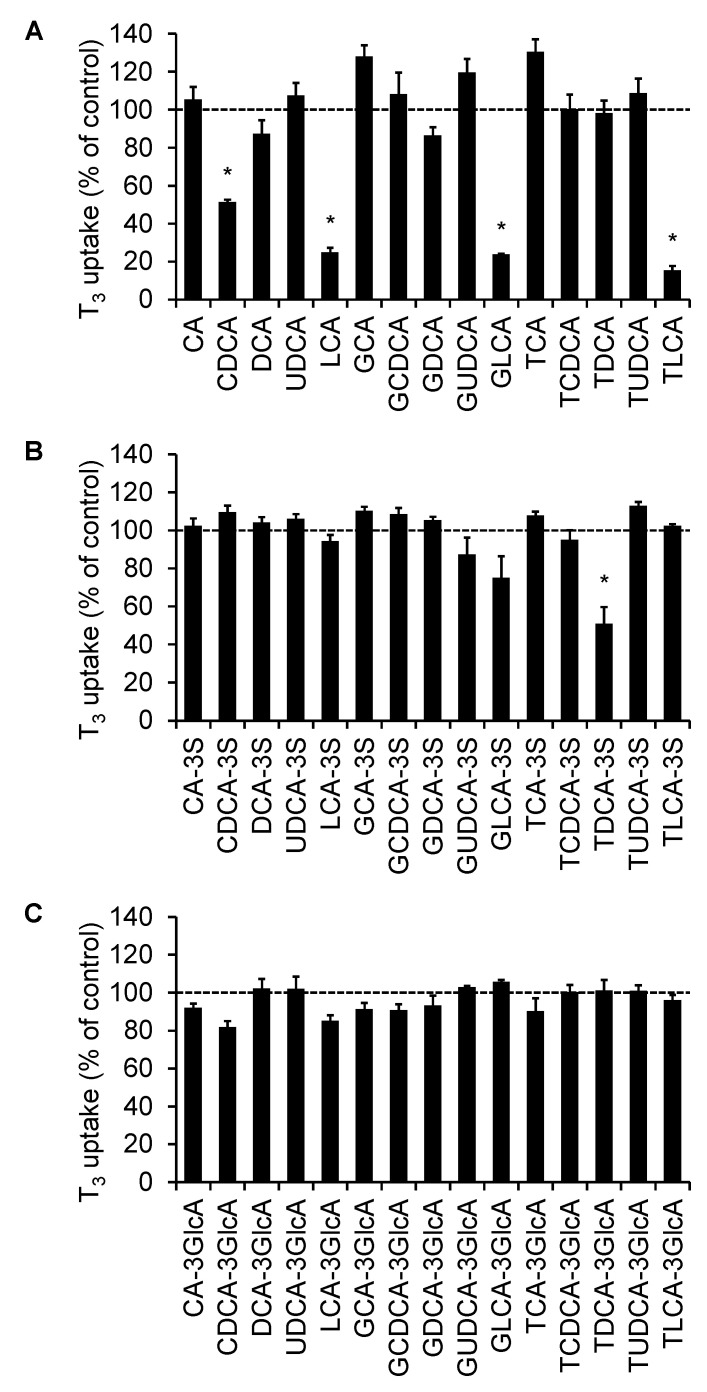
Inhibitory effect of 45 bile acids on OATP4C1-mediated T_3_ transport. (**A**) unconjugated, glycine-conjugated, and taurine-conjugated bile acids, (**B**) bile acid 3-sulfates, and (**C**) bile acid 3-glucuronides. The T_3_ concentration was 1 µM and cells were incubated for 10 min at 37 °C. The bile acid concentrations were 50 µM for CA-3S, LCA-3S, TDCA-3S, LCA-3GlcA, and GLCA-3GlcA, and 100 µM for the other 40 types of bile acids. The OATP4C1-mediated transport was calculated by subtracting the nonspecific uptake of T_3_ by the mock cells from the total cellular uptake by the OATP4C1-expressing cells. Each column and bar represent the mean ± standard error of the mean (*n* = 3). The data are shown as the percentages of transport relative to the control. An asterisk indicates a significant difference from the control by one-way analysis of variance (ANOVA) followed by Tukey’s test (*p* < 0.05).

To determine whether bile acids affected the OATP4C1-mediated transport, OATP4C1-overexpressing cells were used. The effect of 45 bile acids (Figure 1) was measured on T_3_ transport via OATP4C1. The screening concentrations of bile acids were 50 µM for CA-3S, LCA-3S, TDCA-3S, LCA-3-glucuronide (LCA-3GlcA), and GLCA-3GlcA and 100 µM for the other 40 types of bile acids. Although most bile acids did not exhibit inhibitory effects, CDCA (positive control) significantly inhibited the OATP4C1-mediated T_3_ transport by 49.8%. In addition, LCA, GLCA, TLCA, and TDCA-3S showed significant inhibition on the OATP4C1-mediated T_3_ transport (75.2, 76.3, 84.7, and 49.1%, respectively) (Figure 2).

### 2.2. The Concentration-Dependent Inhibitory Effect of Bile Acids

**Figure 3:**
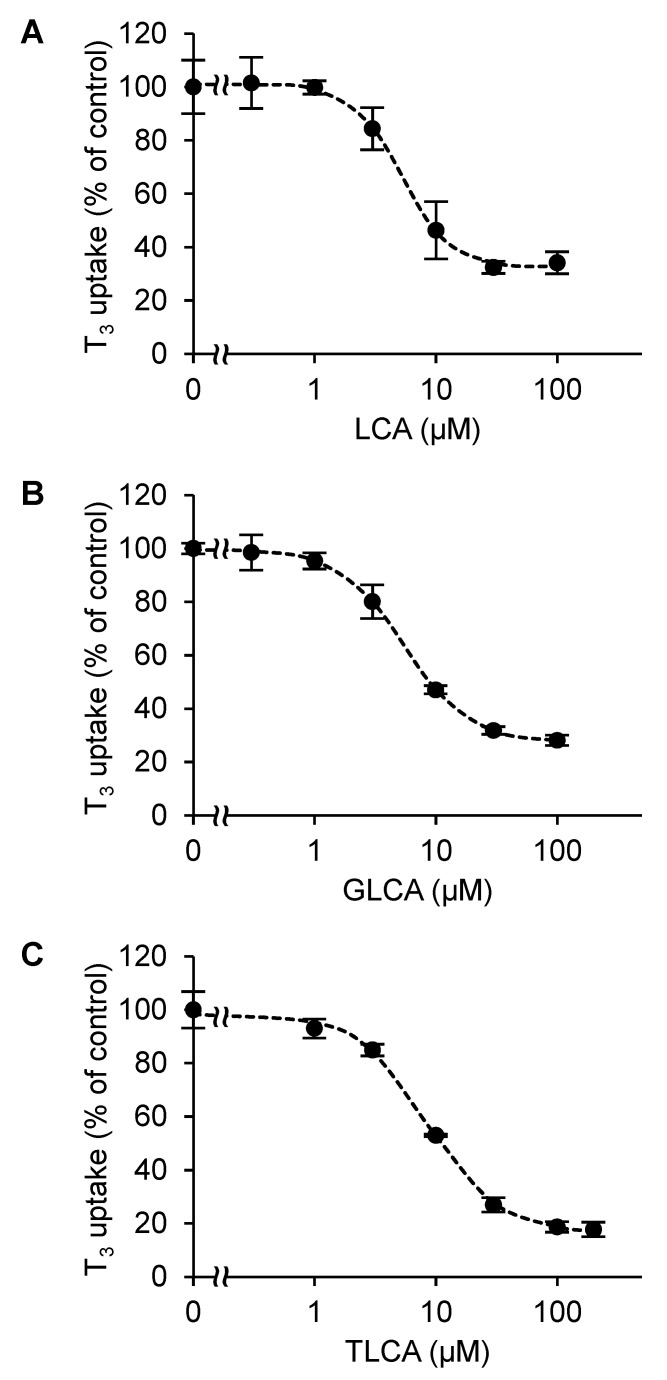
Concentration dependence of the inhibitory effect of the bile acids on the OATP4C1-mediated T_3_ transport. (**A**) LCA, (**B**) GLCA, and (**C**) TLCA. The T_3_ concentration was 1 µM and the cells were incubated for 10 min at 37 °C. The bile acid concentrations were 0, 0.3, 1, 3, 10, 30, and 100 µM for LCA and GLCA, and 0, 1, 3, 10, 30, 100, and 200 µM for TLCA. The OATP4C1-mediated transport was calculated by subtracting the nonspecific uptake of T_3_ by the mock cells from the total cellular uptake by the OATP4C1-expressing cells. Each point and bar represent the mean ± standard error of the mean (*n* = 3). The data are shown as the percentages of transport relative to the control. The dotted lines represent the fitted line obtained by nonlinear least squares regression analysis.

Since LCA, GLCA, and TLCA showed more than 50% inhibition of the OATP4C1-mediated T_3_ transport, we examined whether this inhibitory effect was concentration-dependent. The activity of the transporter was investigated at six different concentrations (0.3, 1, 3, 10, 30, and 100 µM for LCA and GLCA, and 1, 3, 10, 30, 100, and 200 µM for TLCA), and the data were plotted. All these bile acids resulted in a concentration-dependent inhibition of the OATP4C1-mediated T_3_ transport. The inhibition curves were drawn using JMP Pro 16, and the IC_50_ values of LCA, GLCA, and TLCA for OATP4C1 were 6.12 ± 1.21, 9.90 ± 0.475, and 12.3 ± 0.891 µM, respectively (Figure 3). The *K*_i_ values of these bile acids calculated using Equation (1) were 5.23 ± 1.03 µM for LCA, 8.46 ± 0.406 µM for GLCA, and 10.5 ± 0.762 µM for TLCA (Table 1).

### 2.3. Calculation of the BDI Index

To determine the influence of the inhibition of these bile acids on the clinical situation, the BDI indices were calculated by referring to the DDI index using the maximum available concentration of bile acids in the blood. The concentrations of bile acids used to calculate the BDI index are summarized in Table 2. The maximum BDI index of LCA, which showed the strongest inhibition of the OATP4C1-mediated T_3_ transport, was 0.278. Meanwhile, the BDI indices of GLCA and TLCA for the OATP4C1-mediated T_3_ uptake were 0.00982 and 0.00554, respectively (Table 2). LCA showed the highest BDI index for the inhibition of the OATP4C1-mediated T_3_ uptake of the three bile acids; thus, LCA–drug interaction via the renal drug transporter OATP4C1 may occur in patients with liver failure.

### 2.4. Effect of Bile Acids on Cell Viability

**Figure 4:**
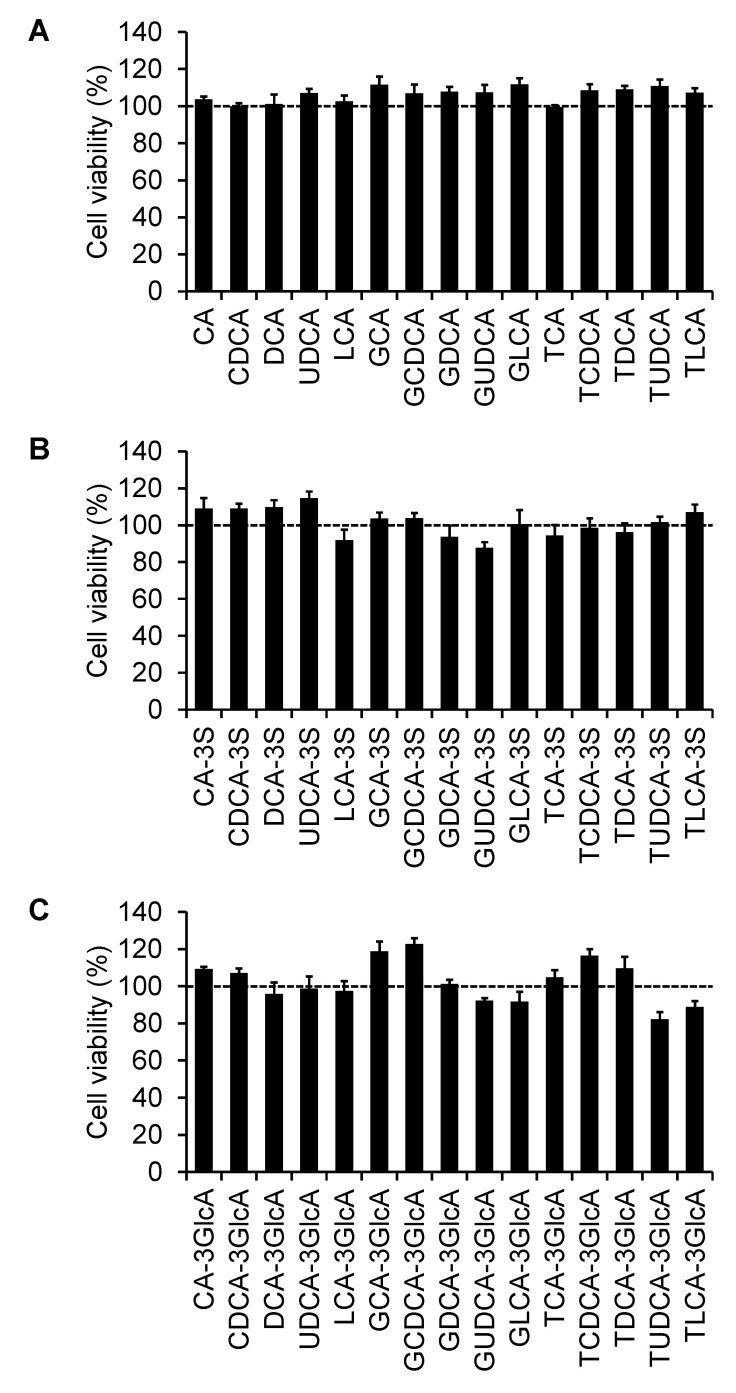
Effect of (**A**) unconjugated, glycine-conjugated, and taurine-conjugated bile acids, (**B**) bile acid 3-sulfates, and (**C**) bile acid 3-glucuronides on cellular viability. The bile acid concentrations were 50 µM for CA-3S, LCA-3S, TDCA-3S, LCA-3GlcA, and GLCA-3GlcA, and 100 µM for the other 40 types of bile acids. The OATP4C1-expressing cells were incubated for 10 min at 37 °C. After a 3 h color reaction, the absorbance was measured at 450 nm. Data are shown as mean ± standard error of the mean (*n* = 5). Data are presented as the percentages of cell viability obtained from the control.

To confirm that the inhibitory effect of bile acids on the OATP4C1-mediated T_3_ transport was not due to cell toxicity, the cellular viability was assessed in the presence of bile acids. The cells were incubated for 10 min with the bile acids using the same concentrations used for screening and the viability was evaluated. The 45 examined bile acids did not affect the cell viability (Figure 4). Moreover, TLCA did not induce cellular toxicity at 200 µM, which was the maximum concentration used in the concentration-dependent study (data not shown). Therefore, the observed decrease in T_3_ transport is due to the direct interaction between bile acids and OATP4C1.

## 3. Discussion

Renal failure affects the dynamics of liver-metabolized drugs by elevating the levels of plasma uremic toxins [2,3]. In liver diseases, bile acids accumulate in the systemic circulation and alter the expression of renal drug transporters that are essential for the excretion of renally excreted drugs [5,6,7,8]. However, the relationship between bile acids and the altered dynamics of renally excreted drugs remains unknown. Bile acids were reported as markers of drug interaction with OATP1B1 and OATP1B3 [28]. Yamaguchi et al. [39] showed that the bile acid CDCA inhibits the renal drug transporter OATP4C1. Based on these reports, we hypothesized that the altered dynamics of renally excreted drugs in patients with liver failure may be due to the BDIs via drug transporters, especially OATP4C1.

In this study, we assessed the effect of bile acids on OATP4C1-mediated transport to elucidate the reason behind the alterations in the dynamics of renally excreted drugs in patients with liver failure. To our knowledge, this is the first report to clarify and summarize the interaction between bile acids and OATP4C1-mediated transport. We screened the effects of 45 types of bile acids on OATP4C1-mediated transport and calculated the IC_50_ and *K*_i_ values of bile acids having an inhibitory effect greater than 50%. We also evaluated the possibility of occurrence of the inhibition of these bile acids in the clinical situation using BDI indices.

First, the effect of 45 types of bile acids on the OATP4C1-mediated transport was investigated; OATP4C1 is the only member of the OATP family that is expressed in the kidneys. The screening results conformed with previous reports, wherein CDCA inhibited the OATP4C1-mediated transport. LCA, GLCA, and TLCA were newly identified as strong inhibitors of the OATP4C1-mediated transport. GLCA and TLCA are the 24-glycine and taurine conjugates of LCA, respectively. On the other hand, the LCA 3-conjugates, namely LCA-3S and LCA-3GlcA, did not inhibit the OATP4C1-mediated transport. Thus, the position and direction of the hydroxyl groups bound to the steroid skeleton could influence the inhibitory effect of bile acids. Next, we evaluated whether the inhibitory effect of bile acids was concentration-dependent. Our results revealed that LCA, GLCA, and TLCA inhibited the OATP4C1-mediated T_3_ transport in a concentration-dependent manner. The IC_50_ values of LCA, GLCA, and TLCA were 6.12, 9.90, and 12.3 µM, respectively, while the *K*_i_ values were 5.23, 8.46, and 10.5 µM, respectively. Although some bile acids induce cell toxicity [43], the inhibitory effects of the bile acids used in this study on the OATP4C1-mediated T_3_ transport were not mediated through decreasing cellular viability.

Thereafter, we determined whether LCA, GLCA, and TLCA inhibited the OATP4C1-mediated T_3_ transport in a clinical setting. Using the blood concentrations of these bile acids of patients with liver disease as a reference, the BDI indices were calculated. For patients with intrahepatic cholestasis of pregnancy, the serum LCA level was 1.7 ± 0.5 µM [40], while the serum GLCA levels were 42.14 ± 133.29 ng/mL (0.0972 µM) in patients with alcoholic liver disease (ALD) [41], 69.6 ± 59.7 nM for patients with severe drug-induced liver injury [44], and 26.80 ± 68.74 ng/mL (0.0618 µM) for patients with primary biliary cirrhosis [41]. Greco et al. [42] reported that the serum TLCA level in patients with post-hepatitis liver cirrhosis was 0.033 ± 0.017 µg/mL (0.0682 µM), while Zhang et al. [45] reported that the plasma TLCA level was 0.020 ± 0.015 µM for patients with cholangiocarcinoma, 0.014 ± 0.013 µM for patients with hepatocellular carcinoma, and 0.010 ± 0.008 µM for patients with gallbladder cancer. Sang et al. [41] reported that the serum TLCA level was 13.63 ± 32.54 ng/mL (0.0282 µM) in patients with ALD. The BDI indices for LCA, GLCA, and TLCA were calculated as 0.278, 0.00982, and 0.00554, respectively. Brites et al. [40] reported that the maximum concentration of serum LCA was 6.0 µM; hence, the BDI index of LCA may reach 0.980. Since 0.1 was suggested as the cutoff value for the DDI index by the regulatory authorities [37], the BDI index for LCA was considered significant, whereas GLCA and TLCA exhibited negligible BDI indices. Meanwhile, the BDI indices obtained in our study were the maximum values, while the IC_50_ values of bile acids were used instead of the *K*_i_ values based on previous reports [37]. Our results showed a slight difference between the *K*_i_ and IC_50_ values since we used a lower concentration of T_3_ in comparison with the *K*_m_ value. Therefore, the BDI indices calculated using the IC_50_ values in this study are reliable. To calculate the exact BDI indices, the protein-binding rates of bile acids should be considered. LCA barely interacts with OATP4C1 under normal conditions in healthy individuals because it binds to serum albumin [46,47,48]. However, the protein-binding rate of drugs in patients with liver failure decreases due to the reduction in albumin synthesis, decline in albumin activity, or conformational changes in albumin [49,50,51]. Hence, the protein-binding rate of LCA may decrease in patients with liver disease. LCA also binds to lipoproteins, such as low-density lipoprotein and high-density lipoprotein [47], which are known to function as carriers of drugs, including antidepressants and antiarrhythmic agents [52]. These drugs may compete for lipoprotein binding under conditions of lower serum albumin levels. Thus, free LCA levels are likely to increase due to the blocking of lipoprotein binding by the unbound drugs, which exist at high levels in patients with liver disease. When the ratio of unbound LCA increases in patients with liver failure, an interaction between LCA and OATP4C1 may occur according to the calculated BDI index. Accordingly, the BDI indices calculated in the present study can be useful for predicting BDIs via OATP4C1 in patients with liver failure. To evaluate the clinical significance of BDI via OATP4C1, an in vivo study using an animal model will be required in the future. Moreover, further in vitro studies to clarify BDI via renal drug transporters (OATs, etc.) are required.

**Figure 5:**
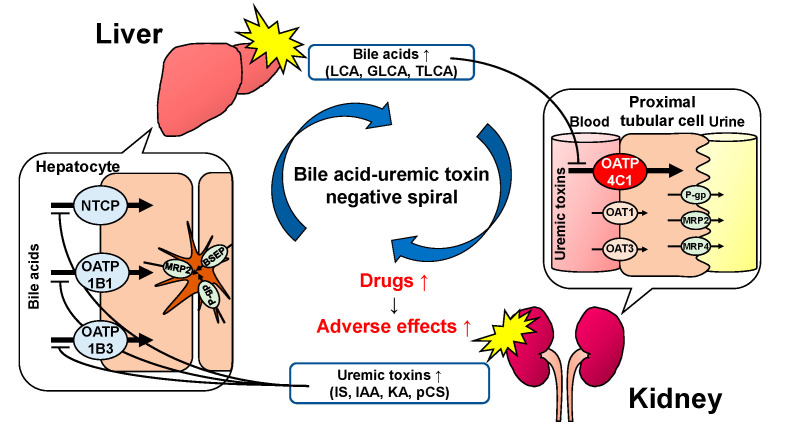
Hypothetical scheme of the “bile acid–uremic toxin negative spiral”. OATP4C1 inhibition by bile acids causes the accumulation of uremic toxins, which may inhibit the bile acid uptake into hepatocytes via NTCP and OATPs. Further accumulation of uremic toxins and bile acids may be induced by the inhibition of OATP4C1 and hepatic bile acid transporters.

OATP4C1 is involved in the excretion of uremic toxins that accumulate in the blood of patients with renal failure [30]. Moreover, renal transporters, including OATP4C1, are downregulated in renal failure [53]. Our previous study [54], in addition to another report [55], revealed that the elevated uremic toxins in the blood inhibited the hepatic transporters OATP1B1, OATP1B3, and NTCP, which regulate the bile acid dynamics. As the present study revealed that the bile acids inhibited the OATP4C1-mediated transport, it may cause the accumulation of uremic toxins. This resulted in the inhibition of bile acid uptake into hepatocytes with a further increase in bile acids in the blood. Thus, the inhibition of OATP4C1 and hepatic bile acid transporters leads to the accumulation of uremic toxins and bile acids. We named this negative spiral as the “bile acid–uremic toxin negative spiral” (Figure 5) that occurs not only in patients with renal failure but also in patients with liver disease. However, further studies are required to confirm this hypothesis.

## 4. Materials and Methods

### 4.1. Materials

Cholic acid (CA), CDCA, and taurine-conjugated lithocholic acid (TLCA) were purchased from Sigma-Aldrich (St. Louis, MO, USA). Deoxycholic acid (DCA) and LCA were purchased from FUJIFILM Wako Pure Chemical Corporation (Osaka, Japan). Triiodothyronine (T_3_), Cell Count Reagent SF, glycine-conjugated ursodeoxycholic acid (GUDCA), taurine-conjugated cholic acid (TCA), ursodeoxycholic acid (UDCA), taurine-conjugated ursodeoxycholic acid (TUDCA), and TCA-3S were purchased from Nacalai Tesque, Inc. (Kyoto, Japan). UDCA-3S was purchased from Alsachim (Illkirch-Graffenstaden, France). Glycine-conjugated cholic acid (GCA), GCDCA, glycine-conjugated deoxycholic acid (GDCA), glycine-conjugated lithocholic acid (GLCA), taurine-conjugated chenodeoxycholic acid (TCDCA), taurine-conjugated deoxycholic acid (TDCA), and GCA-3S were previously synthesized in our laboratory [56]. All the other 27 bile acids, not available in the market, were kindly provided by the Junshin Clinic Bile Acid Institute (Tokyo, Japan). All other chemicals were commercially available and had the highest possible purities.

### 4.2. Cell Culture

MDCKII cells transfected with OATP4C1 or an empty vector were previously established in our laboratory [39]. OATP4C1/MDCKII and mock cells were cultured in Dulbecco’s modified Eagle’s medium supplemented with 10% fetal bovine serum (Gibco^TM^, Thermo Fisher Scientific Inc., Waltham, MA, USA) and G418 (0.5 mg/mL, Nacalai Tesque, Inc., Kyoto, Japan) at 37 °C under 5% CO_2_ and 95% humidified air.

### 4.3. Transport Study

The cellular uptake of T_3_ was measured in monolayer cell cultures grown in 24-well plates. Cells were seeded at a density of 2.0 × 10^5^ cells/well. Thereafter, they were incubated for 24 h in a culture medium containing 5 mM sodium butyrate before the uptake study. The cells were washed once, followed by preincubation in Krebs–Henseleit (KH) buffer (118 mM NaCl, 23.8 mM NaHCO_3_, 4.83 mM KCl, 0.96 mM KH_2_PO_4_, 1.20 mM MgSO_4_, 12.5 mM *N*-(2-hydroxyethyl) piperazine-*N*’-2-ethanesulfonic acid, 5.0 mM D-glucose, and 1.53 mM CaCl_2_; pH 7.4). Cellular uptake was initiated by adding KH buffer containing T_3_ with or without each bile acid. The uptake was terminated after 10 min by replacing the incubation buffer with ice-cold KH buffer. The cells were then washed twice with ice-cold KH buffer. The concentration of T_3_ was measured using liquid chromatography/tandem mass spectrometry. The cellular uptake was presented as the uptake amount divided by the cellular protein amount quantified by the Bradford protein assay. The experiment was performed in triplicate (*n* = 3) and was repeated three times.

All bile acids were dissolved in dimethyl sulfoxide (DMSO) and the final concentration of DMSO was less than 0.5%.

### 4.4. Sample Preparation

The cells were scraped and homogenized in 200 µL of water after the uptake was terminated. Deproteinization was performed by adding equal volumes of acetonitrile containing pravastatin as the internal standard. After vortexing, the mixture was centrifuged at 15,000× *g* for 5 min at 20 °C, and the supernatant was measured.

### 4.5. Liquid Chromatography/Tandem Mass Spectrometry (LC/MS/MS) Condition

LC/MS/MS was used to measure the T_3_ concentrations. For chromatographic separation, a Shimadzu Nexera HPLC System (Shimadzu Corporation, Kyoto, Japan) was used with a Cosmosil 5C18-MS-II column (Nacalai Tesque, Inc.). The mobile phase was water/acetonitrile (70:30, *v*/*v*), containing 0.1% acetic acid at a flow rate of 0.2 mL/min. The column temperature was maintained at 40 °C and the sample injection volume was 5 µL.

Mass spectrometric analysis was performed using an API 5000 (Sciex LLC, Framingham, MA, USA) tandem mass spectrometer. The T_3_ concentration was measured in negative ion mode. Selected reaction monitoring was performed and *m/z* 650 > 127 for T_3_ and *m/z* 423 > 101 for pravastatin were acquired. The collected data were analyzed using Analyst software (version 1.5, SCIEX LLC, Framingham, MA, USA).

### 4.6. Inhibitory Effect of Bile Acids

We investigated the concentration dependence of bile acids, which resulted in more than 50% inhibition of OATP4C1-mediated T_3_ uptake. The inhibition curve was fitted to the Rodbard model. The half-maximum inhibitory concentration (IC_50_) values of the bile acids were calculated using JMP Pro 16 (SAS Institute Inc., Cary, NC, USA). We then acquired the absolute inhibitory constant (*K*_i_) using the following equation (Equation (1)) [57], where [S] is the substrate concentration and *K*_m_ is the Michaelis–Menten constant. The experiment was performed in triplicate (*n* = 3) and was repeated three times.

(1) $$K_{i}=\frac{{\mathrm{IC}}_{50}}{1+\frac{\left[S\right]}{K_{m}}}$$

### 4.7. BDI Index Prediction

The BDI index was calculated using the drug–drug interaction (DDI) index equation [37]. To evaluate the BDI index, we used the IC_50_ values from our in vitro study and the maximum plasma concentration (C_max_) of each bile acid. Then, the possibility of a clinical BDI was predicted to infer the significance of our findings in clinical situations. We used the following equation to calculate the BDI index (Equation (2)) [37].

(2) $$\mathrm{BDI}\;\mathrm{index}=\frac{C_{\max}}{{\mathrm{IC}}_{50}}$$

### 4.8. Effect of Bile Acids on Cell Viability

Cell viability in the presence of bile acids was measured in monolayer cell cultures grown in 96-well plates using Cell Count Reagent SF (Nacalai Tesque, Inc.). Cells were seeded at a density of 1.0 × 10^4^ cells/well, and incubated for 24 h in a culture medium containing 5 mM sodium butyrate before the experiment. After washing, the culture medium was replaced with serum-free culture medium containing bile acid. After 10 min, the incubation was terminated by replacing the medium with Cell Count Reagent SF/culture medium (1:10, *v*/*v*). The color reaction was performed at 37 °C under 5% CO_2_ and 95% humidified air for 3 h and the absorbance was determined at 450 nm using Infinite 200 PRO (TECAN Group Ltd., Männedorf, Switzerland). Absorbance at 600 nm was used as the reference. The experiment was performed in quintuplicate (*n* = 5) and was repeated three times.

### 4.9. Statistical Analysis

Data are expressed as mean ± standard error of the mean. The one-way analysis of variance (ANOVA) followed by Tukey’s test was performed for multiple statistical comparisons. The data were analyzed using JMP Pro 16 (SAS Institute Inc.). Statistical significance was indicated by *p* values less than 0.05.

## 5. Conclusions

Based on the screening results of 45 types of bile acids, LCA, GLCA, and TLCA are newly detected as inhibitors of OATP4C1-mediated T_3_ transport. Dose adjustment of renally excreted drugs may be required in patients with renal failure as well as those with hepatic failure due to the possible interaction of LCA with OATP4C1 in clinical situations. Our findings provide critical information for drug development and drug therapy safety in clinical settings by preventing unanticipated side effects in patients with renal failure as well as in those with hepatic failure.

## Figures and Tables

**Table 1:** IC_50_ and *K*_i_ values of LCA, GLCA, and TLCA.

Bile Acid	IC_50_ (µM)	*K*_i_ (µM)
LCA	6.12 ± 1.21	5.23 ± 1.03
GLCA	9.90 ± 0.475	8.46 ± 0.406
TLCA	12.3 ± 0.891	10.5 ± 0.762

Data are represented as mean ± standard error of the mean.

**Table 2:** BDI indices of LCA, GLCA, and TLCA.

Bile Acid	IC_50_ (µM)	C_max_ (µM)	BDI Index
LCA	6.12 ± 1.21	1.7 [40]	0.278
GLCA	9.90 ± 0.475	0.0972 [41]	0.00982
TLCA	12.3 ± 0.891	0.0682 [42]	0.00554

The IC_50_ values are shown as mean ± standard error of the mean.

## Data Availability

Not applicable.

## Abbreviations

3GlcA, 3-glucuronide; 3S, 3-sulfate; ALD, alcoholic liver disease; BDI, bile acid–drug interaction; CA, cholic acid; CDCA, chenodeoxycholic acid; C_max_, maximum plasma concentration; DCA, deoxycholic acid; DDI, drug–drug interaction; DMSO, dimethyl sulfoxide; GCA, glycine-conjugated cholic acid; GCDCA, glycine-conjugated chenodeoxycholic acid; GDCA, glycine-conjugated deoxycholic acid; GLCA, glycine-conjugated lithocholic acid; GUDCA, glycine-conjugated ursodeoxycholic acid; IC_50_, half-maximum inhibitory concentration; *K*_i_, absolute inhibitory constant; LC/MS/MS, liquid chromatography/tandem mass spectrometry; LCA, lithocholic acid; NTCP, Na^+^-taurocholate cotransporting polypeptide; MRP, multidrug resistance-associated protein; OAT, organic anion transporter; OATP, organic anion-transporting polypeptide; T_3_, triiodothyronine; TCA, taurine-conjugated cholic acid; TCDCA, taurine-conjugated chenodeoxycholic acid; TDCA, taurine-conjugated deoxycholic acid; TLCA, taurine-conjugated lithocholic acid; TUDCA, taurine-conjugated ursodeoxycholic acid; UDCA, ursodeoxycholic acid.
